# Rapid Determination of Kinetic Constants for Slow-Binding Inhibitors and Inactivators of Human Histone Deacetylase 8

**DOI:** 10.3390/ijms25115593

**Published:** 2024-05-21

**Authors:** Aleksandra Kopranovic, Franz-Josef Meyer-Almes

**Affiliations:** Department of Chemical Engineering and Biotechnology, University of Applied Sciences Darmstadt, Haardtring 100, 64295 Darmstadt, Germany

**Keywords:** protein–ligand binding, histone deacetylases, binding mechanism, drug–target interaction

## Abstract

The kinetics and mechanism of drug binding to its target are critical to pharmacological efficacy. A high throughput (HTS) screen often results in hundreds of hits, of which usually only simple IC_50_ values are determined during reconfirmation. However, kinetic parameters such as residence time for reversible inhibitors and the *k_inact_*/*K_I_* ratio, which is the critical measure for evaluating covalent inactivators, are early predictive measures to assess the chances of success of the hits in the clinic. Using the promising cancer target human histone deacetylase 8 as an example, we present a robust method that calculates concentration-dependent apparent rate constants for the inhibition or inactivation of HDAC8 from dose–response curves recorded after different pre-incubation times. With these data, hit compounds can be classified according to their mechanism of action, and the relevant kinetic parameters can be calculated in a highly parallel fashion. HDAC8 inhibitors with known modes of action were correctly assigned to their mechanism, and the binding mechanisms of some hits from an internal HDAC8 screening campaign were newly determined. The oxonitriles SVE04 and SVE27 were classified as fast reversible HDAC8 inhibitors with moderate time-constant IC_50_ values of 4.2 and 2.6 µM, respectively. The hit compound TJ-19-24 and SAH03 behave like slow two-step inactivators or reversible inhibitors, with a very low reverse isomerization rate.

## 1. Introduction

The family of histone deacetylases (HDACs) is an important example of enzymes that are involved in many important cellular processes, such as transcription, cellular metabolism, and cell cycles, and have therefore become promising targets in the indication areas of cancer, neurodegenerative diseases, cardiovascular diseases, diabetes, immunological disorders, and infectious diseases [1,2,3,4,5]. The currently clinically approved HDAC inhibitors inhibit several isozymes at the same time, as the active centers of all zinc-dependent HDACs are sequentially and structurally highly conserved [6]. To achieve selectivity, small side pockets or regions on the enzyme surface have been exploited in order to achieve the best possible affinity to a specific HDAC [7,8,9,10,11]. Interestingly, several HDACi have been developed that owe their isoenzyme selectivity to slower dissociation kinetics for a specific HDAC [12,13].

The binding kinetics between drugs and targets determines the interface between pharmacokinetics and pharmacodynamics. Longer occupancy times of a drug on a target enable lower dosing and longer duration of the pharmacological effect, since the drug first has to dissociate from the drug–target complex before it is metabolized or excreted. Therefore, the drug–target residence time (RT), defined as the reciprocal overall dissociation rate constant of a drug–target complex, has been proposed as an important predictor of pharmocodynamic activity, whereas drugs with longer RT are associated with higher efficacy and fewer side effects [14,15,16,17]. Slow-binding inhibitors and irreversible inactivators of target enzymes are of increasing importance as effective therapeutics against many diseases, especially cancer and bacterial or viral infections. This can be seen from the fact that newly approved drugs usually have longer RTs than first-generation drugs against the same target. For example, HIV protease indinavir, which was approved in 1996, has an RT of 0.3 h, whereas the RT of darunavir, approved in 2006, has been determined to be 247 h [18]. The increased RT correlated with a higher mutational barrier for the development of resistance against anti-HIV medication [18]. Drug RT is of particular importance for the development of kinase inhibitors. The number of FDA-approved drugs with prolonged RTs has increased over time. For example, EGFR inhibitors gefitinib and erlotinib, approved in 2003 and 2004, respectively, have RTs < 10 min. In contrast, the 2007-approved lapatinib has a significantly longer RT of 7.2 h. In a comprehensive study, Georgi et al. analyzed the dissociation rates of 270 kinase inhibitors against 40 pharmacologically relevant protein kinases and essentially found that both association and dissociation rates contribute to in vivo efficacy [19]. However, the enrichment of chemical entities with slow dissociation rates among approved drugs suggests an important role regarding clinical success [20,21,22,23]. Similar trends with bacterial targets confirm that slow-binding inhibition kinetics translate into in vivo effects [24,25]. However, in other cases, toxic effects may be incurred from too-long target occupation, and shorter RTs may be more desirable. For instance, long RT was believed to contribute to the severe side effects of the antipsychotic drug haloperidol, which is a dopamine D2 receptor antagonist [26]. Longer target engagement may be particularly advantageous for anticancer drugs, leading to the development of covalent inactivators of protein kinases, which permanently occupy their target. Second-generation tyrosine kinase inhibitors that bind covalently show promising outcomes in the treatment of non-small-cell lung and breast cancer. Ibrutinib and osimertinib are representative examples of covalent inactivators of Bruton tyrosine kinase (BTK) for the treatment of mantle cell lymphoma and chronic lymphocytic leukemia and epidermal growth factor receptor (EGFR) for the treatment of non-small-cell lung cancer, respectively [27,28]. There are also covalent drugs against other target classes, such as sotorasib (KRAS), nirmatrelvir (SARS-CoV-2 protease), and voxelotor (sickle cell β-hemoglobin) [14]. Twenty-five clinical trials are recruiting, enrolling, or active [29].

Given that the elucidation of the kinetic and mechanistic properties of drug candidates represents an increasingly crucial aspect of medicinal chemistry optimization, there emerges a pressing necessity for the implementation of efficacious high-throughput methodologies to ascertain the kinetic constants associated with non-covalent inhibitor and irreversible inactivator interactions with target proteins. Common biophysical methods for determining the binding kinetics of drugs to target proteins include stopped-flow, surface plasmon resonance (SPR), and isothermal titration calorimetry (ITC) [18,30,31]. These methods have limited throughput, which only allows the mechanistic characterization of a few substances. Fluorescence-based displacement assays allow a higher throughput but require a suitable fluorescently labeled tracer with favorable binding kinetics [32].

For enzymes, effective methods have been described that allow the determination of kinetic parameters for drug–target interaction based on continuous or discontinuous activity assays using chromogenic or fluorogenic substrates [33]. The analysis requires that the product formation of the uninhibited enzyme reaction is linear over a time range of several hours, which is not easy to achieve in practice because the protein is not always stable for this long or the substrate is consumed. A useful method to measure the kinetics of covalent bond formation is mass spectrometry, which allows assessment of the modification kinetics of particular amino acids [34]. However, this method is quite complex, and a simpler and more robust approach is required to determine the kinetic inactivation parameters in a high throughput mode. An interesting method for the determination of kinetic constants for the irreversible inhibition of enzymes was reported by Krippendorff et al. [35]. Their method allows the direct determination of the inactivation rate constant, *k_inact_*, and *K_I_*, which is equal to the inhibitor concentration with half-maximum *k_inact_*, from time-dependent IC_50_ values. The *k_inact_*/*K_I_* ratio is considered as the most important metric to interpret structure relationships (SARs) and optimize selective covalent inactivators by medicinal chemistry. However, the data analysis requires the complicated solution of an implicit equation and turns out to be relatively unstable in practice, as it depends very sensitively on the assumed mechanism, the effective substrate concentration, and the associated Michaelis constant *K_M_*.

Here, we apply a very simple and robust high-throughput method for the determination of kinetic constants of HDAC8 inhibitors and inactivators based on dose response curves after different pre-incubation times. The method allows a rapid categorization of fast/slow-binding non-covalent inhibitors or covalent inactivators with respect to their kinetics and binding mechanism. In contrast to the method of Krippendorff et al., no assumption about the binding mechanism is necessary. In the special embodiment, possible linearity problems of the product formation kinetics due to substrate consumption are prevented by the addition of fresh substrate after defined pre-incubation times and very short reaction times. Protein instability can be greatly corrected by referencing. This method can be easily transferred to other enzymes.

## 2. Results

General binding mechanism of inhibitory compounds to a target protein include the following chemical reaction equations:

Reversible binding:

(1) $$P+I\overset{k_{on}}{\underset{k_{off}}{⇄}}PI$$

(2) $$P+I\overset{k_{on}}{\underset{k_{off}}{⇄}}PI\overset{k_{r}}{\underset{k_{-r}}{⇄}}PI^{*}$$

Covalent inactivation:

(3) $$P+I\overset{k_{inact}}{\to }P-I$$

(4) $$P+I\overset{k_{on}}{\underset{k_{off}}{⇄}}PI\overset{k_{inact}}{\to }P-I$$

**Figure 1:**
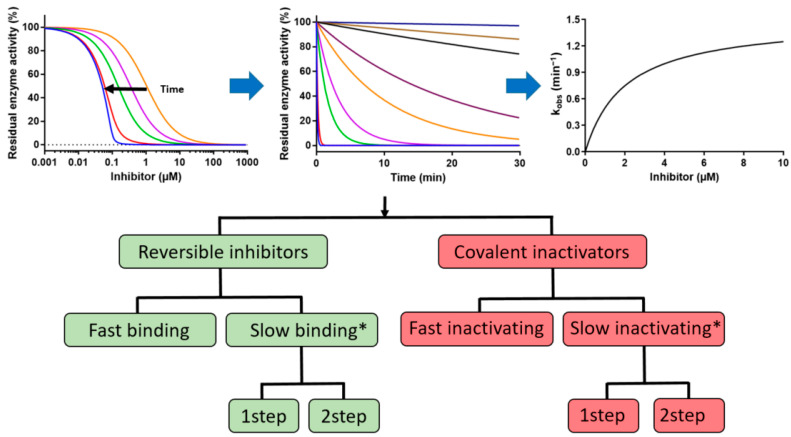
Scheme of procedure for the classification of inhibitory mode of action using time-dependent dose–response curves of enzyme activity assays. Analysis of the data enables the classification into sub-groups as shown in the lower part. * Indicates that reversible inhibitors with very slow reverse enzyme isomerization rate constant *k_−r_* cannot be distinguished from covalent inactivators by this type of analysis.

We propose a simple procedure to categorize the interaction of a target protein and a ligand with respect to their above-mentioned interaction mechanisms (Figure 1). For this approach, an enzyme activity assay is required, which enables continuous measurement of substrate turnover. Alternatively, the enzyme reaction can be started after a defined pre-incubation time with the inhibitor by adding substrate. In this case, the enzyme activity has to be determined very quickly in comparison to the inactivation kinetics, which is usually in the minutes to hours range. The procedure starts with time-dependent dose–response curves, which are converted into decay curves of enzyme activity in the presence of different inhibitor or inactivator concentrations. In the last step, *k_obs_* is plotted versus [*I*] and analyzed using a suitable Equations (8)–(11).

To demonstrate the general suitability of our approach to analyze kinetic data sets of slow enzyme inhibition, we first reanalyzed data from the literature. For that purpose, we selected experimental data of particularly high quality that showed the inactivation kinetics of CYP enzymes (Figure S1, Table 1). Data were analyzed assuming one-step or two-step inactivation mechanisms according to the reaction mechanisms described in Equations (3) and (4). Firstly, the remaining enzyme activities were extracted from the data sets for all inactivator concentrations and plotted against the respective preincubation times used. Secondly, the resulting decay curves for different inactivator concentrations were fitted to mono-exponential functions, thus yielding observed inactivation rates *k_obs_* for all inactivator concentrations. Thirdly, *k_obs_* was plotted against the inactivator concentration and fitted to a Michaelis–Menten-like saturation function for the general two-step inactivation mechanism (Equation (11)).

This equation simplifies to *k_obs_* = *k_inact_*/*K_I_* [*I*], where *K_I_* is much greater than the inhibitor concentration [*I*], resulting in a linear relationship with slope *k_inact_*/*K_I_*. This situation is indistinguishable from a one-step inactivation mechanism and was observed for fluoxetine, whereas the other investigated inactivators reacted according to a two-step mechanism (Table 1, Figure S1). The reanalysis of the literature data using the above-mentioned procedure provided very good agreement with the published *k_inact_*/*K_I_* parameters, demonstrating the simplicity, robustness, and suitability of this approach to calculate meaningful key parameters to evaluate inhibitors and categorize them according to their kinetics.

### 2.1. Reversible HDAC8 Inhibitors

**Figure 2:**
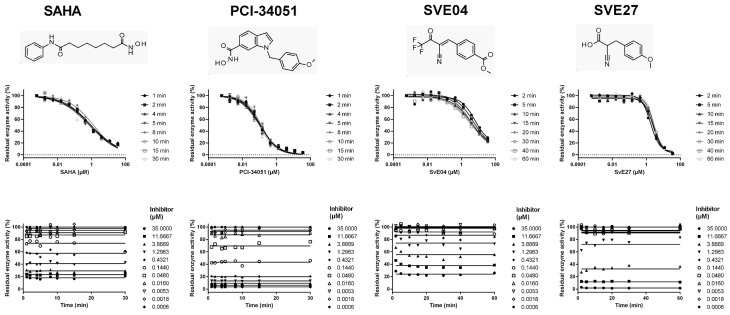
Dose–response curves and constant HDAC8 activity in the presence of fast reversible HDAC8 inhibitors after different indicated pre-incubation times.

In the next step, we applied the procedure for the determination of kinetic parameters to mechanistically different types of fast-/slow-binding reversible inhibitors, as well as covalent inactivators of HDAC8 (Figure 2). Essentially, time-dependent dose–response curves using 10 different compound concentrations were measured after different pre-incubation times. At first, known fast reversible inhibitors like the clinically approved vorinostat (suberoylanilide hydroxamic acid, SAHA), a pan-HDAC inhibitor, and reference compound PCI-34051, an established highly selective HDAC8 inhibitor, were tested. The rapid adjustment of the chemical equilibrium was confirmed by matching dose–response curves after different pre-incubation times (Figure 2).

**Figure 3:**
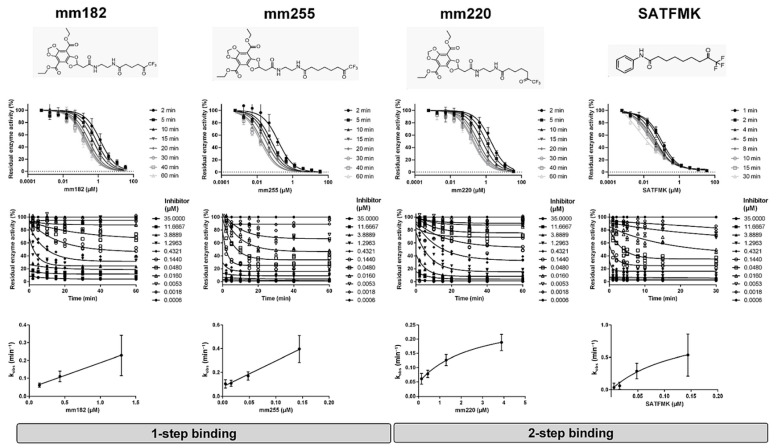
Dose–response curves, concentration-dependent decay of residual of enzyme activity, and observed inhibition rates of *k_obs_* in the presence of slow-binding reversible trifluoromethylketone HDAC8 inhibitors after different indicated pre-incubation times.

Notably, chemical equilibrium was achieved after less than 2 min, which corresponds to an observed rate constant *k_obs_* > 0.5 min^−1^. We also tested oxonitrile compounds SVE04 and SVE27, which emerged as hits in an internal HDAC8 screening campaign, for their binding kinetics to HDAC8, and found that SVE04 and SVE27 clearly showed fast reversible binding with medium micromolar activities (Figure 2, Table 2). Next, we tested several trifluoromethylketone analogs, mm182, mm220, and mm255. These [1,3]dioxolo[4,5-f]benzodioxole conjugates with different linker lengths and a trifluoromethylketone warhead for zinc chelation were originally developed as fluorescence probes for competitive HDAC-binding assays [40]. The compounds turned out to be slow-binding reversible ligands of HDAC8 with one or two binding steps, as previously determined in fluorescence polarization binding assays [12]. The slow binding kinetics was confirmed by using the enzyme activity-based method in this study (Figure 3); mm183 and mm255 were categorized as slow reversible one-step binders, whereas mm220 clearly showed two-step binding behavior. Notably, the *y*-axis intercept in the plot *k_obs_* vs. inhibitor concentration was clearly different from zero, demonstrating reversible binding behavior (Figure 3). SATFMK was previously characterized as a very potent reversible slow-binding inhibitor of HDAC8 using a fluorescence resonance energy transfer (FRET)-based displacement binding assay [41]. The inhibitor behavior was consistent with two binding steps using the time-dependent method of this study. However, the curvature of the curve is not clear and could also indicate a slow reversible one-step binding. In this case, the *y*-axis intercept was very small and could not be distinguished from zero. This finding implies either very slow reverse isomerization (very low *k_−r_*) of the final complex (Equation (2)), a slow off-rate in the case of a one-step mechanism or covalent inactivation of HDAC8 with no dissociation of the final complex (Equation (4)). The thermodynamic and kinetic constants for the interaction of reversible inhibitors with HDAC8 are summarized in Table 2.

### 2.2. Irreversible Covalent Inactivators

Several further compounds with unusual structures arose from our HDAC8 screen. Among the best hits were compounds with known cysteine reactivity, for example, ebselen, NEM, or (4-carboxyphenyl)-chloro-mercury. Other potent hits with cysteine reactivity were found, which on first sight would not chemically react with HDAC8. One of these cases was P2742. Inspecting the literature revealed that the compound would decompose in thiophenol, which might form a mixed disulfide under oxidative conditions with one of cysteines in HDAC8. A thorough investigation of the thiol reactivity of P2742 revealed a highly complex reaction mechanism, which eventually leads to the cyanylation of Cys153 in the binding pocket of HDAC8 [44]. Also, P2742 induces several intramolecular disulfide bridges in HDAC8, which deactivate the enzyme [45]. Thus, P2742 was considered to be a very efficient inactivator of HDAC8.

**Figure 4:**
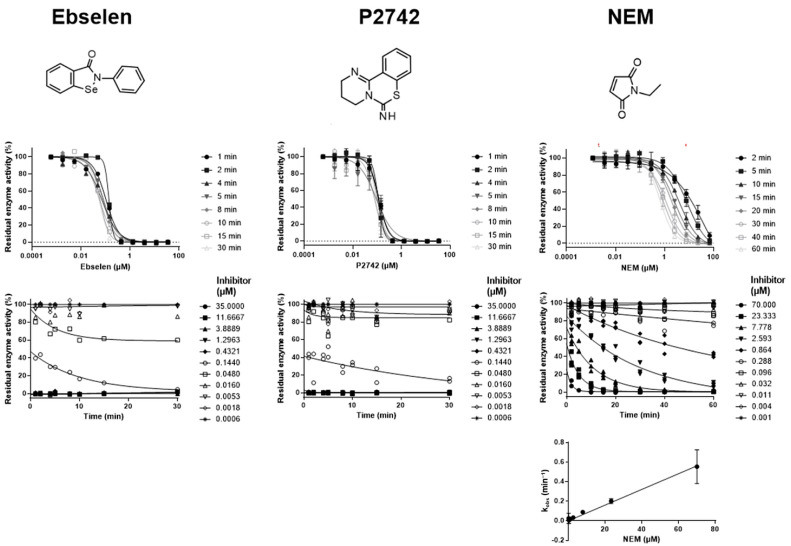
Dose–response curves and concentration-dependent decay of residual enzyme activity in the presence of fast (ebselen and P2742) and slow one-step (NEM) HDAC8 inactivators after different indicated pre-incubation times.

When looking at the time-dependent dose–response curves of ebselen and P2742, it is striking that there is no major shift (Figure 4). Furthermore, the curves are very steep, indicating a high hill factor, and the inflection point of the curves is close to the concentration of HDAC8 used in the assay. Around the inflection point, only one or two concentrations (out of ten) were observed that indicated a time-dependent process. However, the data were inaccurate in the steep descent of the curve and did not allow a more precise kinetic analysis. Collectively, the data indicated that the compounds rapidly inactivated HDAC8 and essentially titrated it. Here too, the inactivation rate, *k_inact_*, cannot be precisely determined due to the time limitation of the assay, but can only be estimated to be greater than 2 min^−1^. In contrast, the known cysteine-reactive N-ethylmaleimide (NEM) is a slow-inactivating compound to HDAC8, indicated by a slow shift of the dose–response curve over time towards smaller apparent IC_50_ values (Figure 4). *k_obs_* for the inactivation reaction is linearly dependent on the NEM concentration up to 23 µM, and a linear fit passes through the origin, which is characteristic for unspecific one-step affinity labeling. However, a slight curvature at higher concentrations than 60 µM cannot be completely ruled out due to experimental error. We also analyzed TJ19-28, which was previously reported as an effective cysteine modifier and also as inactivating HDAC8 [34]. Analysis of the slow time-dependent shift of the dose–response curves with increasing pre-incubation time revealed that *k_obs_* was a hyperbolic function of the compound concentration with zero *y*-axis intercept, consistent with a two-step inactivation mechanism.

**Figure 5:**
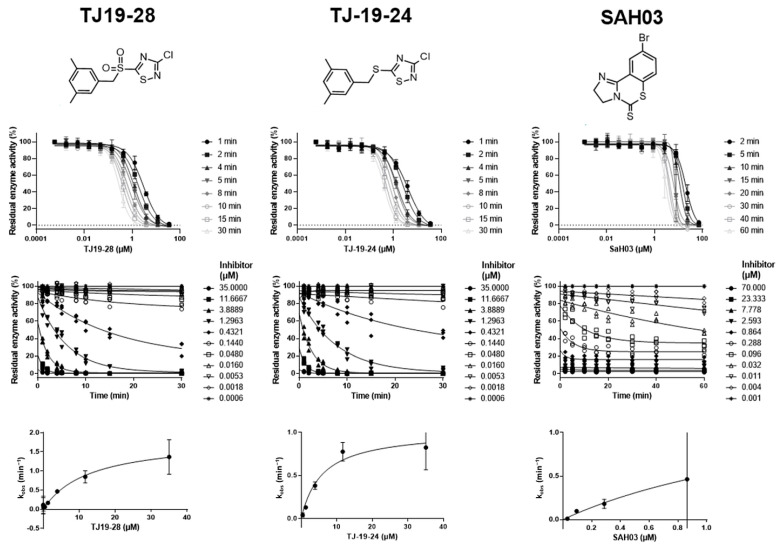
Dose–response curves, concentration-dependent decay of residual of HDAC8 activity, and observed inactivation rates of *k_obs_* in the presence of slow two-step inactivators after different indicated pre-incubation times.

For comparison, we tested TJ-19-24, where the sulfonyl group was replaced by a sulfanyl group (Figure 5). It has previously been shown that this substitution results in a loss of thiol reactivity against 5-thio-2-nitrobenzoic acid as the reaction partner [34]. It was therefore surprising to observe that TJ-19-24 behaved in the same way as a slow covalent inactivator. This finding is discussed in detail later in the paper. We also tested the benzothiazine-thione SAH03, which was reported as a selective HDAC8 inhibitor and does not react with gluthatione [46]. The observed inhibition rate constant, *k_obs_*, was a hyperbolic function of the compound concentration, with a *y*-axis intercept of zero indicating a slow two-step inactivation of HDAC8. The calculated kinetic parameters are summarized in Table 3. The *k_inact_*/*K_I_* ratio is the key parameter for medicinal chemists who want to optimize covalent drug candidates. One-step inactivators like NEM are usually unspecific thiol modifiers. In contrast, the TJ- compounds and SAH03 showed a two-step mechanism, suggesting that the non-covalent formation of the encounter complex with HDAC8 is important for the selective recognition and effective inactivation of the target enzyme. The rate constant *k_inact_* was similar for all three two-step inactivators, but K_I_ varied more, ultimately resulting in SAH03 having the best *k_inact_*/*K_I_* ratio of 12,500 M^−1^s^−1^.

## 3. Discussion

Continuous activity assays are frequently used to determine the inhibition kinetics of active substances on target enzymes [33]. For correct data analysis, however, the progress curves must usually be linear over a period of at least 30–60 min, which is difficult to achieve in reality because the enzymes are either not stable or substrate consumption becomes noticeable. Assuming a two-step inactivation mechanism as described in Equation (4), Krippendorff et al. developed a complex equation that describes the dependence of the IC_50_ value for the inactivation of an enzyme on time and that can calculate both *k_inact_* and *K_I_* via a data fit [35]. However, this approach does not allow other mechanisms to be taken into account. The data fitting is also complex, as an implicit equation has to be fitted, which also depends very sensitively on the Michaelis constant *K_M_*. Here, we solve the aforementioned challenges by carrying out a discontinuous enzyme activity assay, whereby the enzyme is pre-incubated for different times in the presence of 10 different compound concentrations. After the specified incubation periods, the enzymatic activity of HDAC8 was assessed in a rapid reaction, which was important for achieving the highest possible time resolution. The format for the assay allows the parallel testing of eight different inhibitors on a 96-well microtiter plate. A slow decrease in enzyme activity over a longer period of time can be largely corrected by referring to an uninhibited control. It was important to optimize the assay so that a good signal-to-noise ratio was obtained after a reaction time of 1–2 min in order to resolve relatively fast kinetics in time. However, the enzyme concentration was not allowed to become too high so that IC_50_ values of more potent substances could still be reasonably determined. As a compromise, we used 100 nM HDAC8, as the good classification of the inhibitors with regard to the inhibition mechanism was more important to us than the precise determination of the IC_50_ values of highly potent substances. Using this optimized method, we were able to distinguish very well between fast and slow reversible inhibition and irreversible inactivation mechanisms. In the case of slow enzyme inhibition, it was also possible to distinguish reliably between one-step and two-step mechanisms. In our assay setup, only kinetics whose *k_obs_* were approximately 2 min^−1^ or lower could be resolved in time. We confirmed SAHA and PCI-34051 as known fast-reversible inhibitors. This was evident from the fact that the dose–response curves did not change over time (Figure 2). However, the previously determined IC_50_ value of PCI-34051 was lower than the HDAC8 concentration used because of very tight binding, which was similar to a quantitative titration of the enzyme activity. Consequently, for PCI-34051, no clear distinction could be made between fast-reversible tight binding or irreversible inactivation using our approach. The known fast covalent inactivators ebselen and P2742 behave similarly (Figure 4). The binding mechanism of the oxonitrils SVE04 and SVE27 has not been previously analyzed. The compounds were clearly classified as fast-reversible inhibitors because their dose–response curves did not change over time and their IC_50_ values were significantly higher than the enzyme concentration used (Figure 2). The trifluoromethylketones mm182, mm220, and mm255 were clearly identified as reversible slow-binding inhibitors. For cases of medium to slow enzyme inhibition, reversibility can be immediately observed from a non-zero value of the *y*-axis intercept in a *k_obs_* vs. [*I*] plot (Figure 3). SATFMK also showed a slow shift in the dose–response curve over time. However, the *y*-axis intercept was not distinguishable from zero and thus from an irreversible inactivation mechanism. In such a case, additional experiments, for example, rapid dilution or dialysis experiments, have to be performed to enforce and measure dissociation after a prolonged time. In the case of SATFMK, the very small *y*-axis intercept is actually due to its very high binding affinity, with a K_d_ of 5.6 nM. The reverse isomerization rate, *k_−r_*, was previously determined to be 0.000355 s^−1^ or 0.021 min^−1^ using a FRET-based displacement assay [41]. Due to the characteristic linear or hyperbolic dependence of *k_obs_* on the inhibitor concentration, mm182 and mm255 could be assigned a one-step mechanism, and mm220 and SATFMK a two-step mechanism. NEM, a known cysteine modifier, showed a slow inactivation behavior, characterized by a temporal shift of the dose–response curves to lower inactivator concentrations, a linear relationship in the *k_obs_* vs. [*I*] plot, and a data fit passing through the origin (Figure 4). The linear *k_obs_* curve is typical for non-specific labelling. The bimolecular reaction rate for NEM inactivating HDAC8 was determined to be 133 M^−1^s^−1^, which is lower than the rate constant for the reaction of NEM with cysteine at pH 8 (*k_inact_* = 15,300 M^−1^s^−1^) as determined by Gorin et al. [48]. However, it is important to note that the rate constant with thiol-containing compounds is strongly dependent on pH. Additionally, the pKs value of the thiol in different chemical microenvironments of proteins may vary significantly [49]. For example, it was found that the reaction rate of NEM with 1-amino-2-methyl-2-propanethiol at a pH of 7.0 had a rate constant more than 100 times lower than that of cysteine [48]. TJ19-28 has been described as a cysteine-reactive agent that also inactivates HDAC8. Here, we confirmed that the compound reacts according to an irreversible inactivation mechanism. The hyperbolic dependence of *k_obs_* on [*I*] suggested a two-step mechanism, in which a non-covalent encounter complex precedes the inactivation reaction (Equation (4)). The analogue TJ-19-24 differs from TJ19-28 only in the replacement of the sulfonyl by a sulfanyl group. This eliminates the reactivity against the thiol model compound 5-thio-2-nitrobenzoic acid [34]. Surprisingly, TJ-19-24 showed a slow two-step inactivation like TJ19-28 in our study. This apparent contradiction could be explained by the fact that TJ-19-24 could be orientated in the binding pocket of HDAC8 in such a way that the spatial conditions and chemical microenvironment allow a reaction with a nucleophilic amino acid. Similarly, it was previously shown for SAH03 that the substance is resistant to gluthathione [46]. But SAH03 unexpectedly also showed a two-step inactivation mechanism against HDAC8. As previously discussed for SATFMK, which dissociates very slowly from HDAC8, further experiments are needed to clarify whether the *y*-axis intercept in the *k_obs_* vs. [*I*] plot for TJ-19-24 and SAH03 was due to very slow dissociation or covalent inactivation. Overall, it was shown that the presented method is very robust, has a high throughput, and allows a rapid classification of HDAC8 inhibitors by the mechanism of action. In addition, suitable data fits allowed the calculation of kinetic parameters for slow inhibitors or inactivators.

## 4. Materials and Methods

### 4.1. Materials

Inhibitors were purchased at the following companies: SAHA, Ebselen, PCI 34051 were purchased from Cayman chemical company (Ann Arbor, MI, USA), SVE04 and SVE27 from Santa Cruz Biotechnology (Dallas, TX, USA), P2724 from Tocris bioscience (Bristol, UK), and N-Ethylmaleimide from Sigma-Aldrich/Merck (Darmstadt, Germany). Recombinant human HDAC8 was produced as previously described [44].

### 4.2. Determining Time-Dependent IC_50_ Values

HDAC8 activity was determined using a fluorometric assay, as previously described [44]. In short, HDAC8 (100 nM) was incubated with a serial dilution of the compounds for the indicated times at 30 °C in HDAC8 assay buffer (25 mM Tris-HCl, 75 mM KCl, and 0.001% Pluronic F-127, pH 8.0). The enzyme reaction was initiated by adding 20 μM of the substrate Boc-Lys(TFA)-AMC (Bachem, Bubendorf, Switzerland). After 5 min, the reaction was stopped, and a fluorescence signal was developed by adding 83 µM SAHA and 0.42 mg/mL trypsin. The measurements were carried out using a fluorescence microplate reader (PHERAstar FS, BMG LABTECH, Ortenberg, Germany) with fluorescence excitation at 360 nm and emission at 460 nm. IC_50_ values were calculated using GraphPad Prism 9 by generating dose–response curves and fitting them to a 4-parameter fit model [50]. The results represent the mean ± SD of three independent experiments.

### 4.3. Calculation of Binding Constants and Kinetic Parameters

The binding constant *K_i_* for the 1-step binding mechanism in Equation (5) was calculated as follows:

(5) $$K_{i}=\frac{k_{off}}{k_{on}}$$

IC_50_ values were converted into *K_i_* values according to Cheng and Prusoff [51], with a Michaelis constant, *K_M_*, of 8.9 µM and an applied substrate concentration, [*S*], of 20 µM.

(6) $$K_{i}=\frac{{IC}_{50}}{\left(1+\frac{\left[S\right]}{K_{M}}\right)}$$

Apparent rate constants, *k_obs_*, for slow inhibition of HDAC8 activity were obtained by fitting the time-dependent decrease of enzyme activity in the presence of inhibitors or inactivators to a one-exponential decay function:

(7) $$HDAC8\left(active\right)=100\%*\exp(-k_{obs}\;t)$$

A plot of *k_obs_* versus inhibitor or inactivator concentration ([*I*]) allows distinguishing between 1-step or 2-step behavior: a straight line indicates 1-step, and a saturation curve indicates a 2-step mechanism. Kinetic parameters were obtained from fitting *k_obs_* vs. [*I*] data to the following functions for the indicated mechanism:

Reversible 1-step binding (see Equation (1)):

(8) $$k_{obs}=k_{on}[I]+k_{off}$$

where *k_on_* and *k_off_* are association and dissociation rate constants, respectively.

Reversible 2-step binding (see Equation (2)):

(9) $$k_{obs}=k_{-r}+\left[\frac{k_{r}}{1+\frac{{IC}_{50}}{\left[I\right]}}\right]$$

where *k_r_* and *k*_−*r*_ are forward and reverse isomerization rate constants of the second step.

Irreversible 1-step inactivation (see Equation (3)):

(10) $$k_{obs}=\frac{k_{inact}}{K_{I}}[I]$$

where *k_inact_* is the inactivation rate, *K_I_* is [*I*], and *k_inact_* is the half-maximum.

Irreversible 2-step inactivation (see Equation (4)):

(11) $$k_{obs}=\frac{k_{inact}[I]}{K_{I\;}+[I]}$$

## 5. Conclusions

Drug–target binding combines pharmacokinetics and pharmacodynamics. The kinetics and mechanism of this interaction determine how long an active substance remains on its target protein and how long it remains effective until it is metabolized or excreted. The specific mechanism of action of a drug can include conformational changes of drug–protein complexes, which then enable further interactions with other proteins or biologically relevant molecules in the cell. In this way, a specific binding mechanism can trigger a specific biological response that cannot be achieved in another way. In order to increase the probability of clinical success, it is extremely important to identify those compounds with the most promising binding mechanisms at an early stage of drug discovery and to at least roughly categorize them into classes of different mechanisms. This requires a method enabling a high throughput. Ideally, important parameters such as RT for slow-reversible inhibitors or the *k_inact_*/*K_I_* ratio, which is crucial for the evaluation of covalent inactivators, can be calculated directly from the data. In this study, we present a simple and robust method for the high-throughput testing of substances that inhibit the enzyme activity of HDAC8. Using this method, we showed that substances with known inhibitory modes of action were correctly assigned. For some compounds, the binding mechanism and kinetic parameters were determined for the first time. As with other methods, it is difficult to distinguish substances with extremely long RT from irreversible inactivators. However, such cases can be clarified by straightforward follow-up experiments. This makes it possible to classify hundreds of hits from an HTS screening campaign according to the mechanism of action. The critical kinetic parameters can be calculated directly from the data, allowing the compounds to be evaluated in detail at an early stage of drug discovery.

## Figures and Tables

**Table 1:** Kinetic constants for the inactivation of CYP enzymes. Comparison of literature and reanalyzed data (Figure S1).

	Literature		Reanalysis	
Substance	*k_inact_* (min^−1^)	*K_I_* (µM)	*k_inact_* (min^−1^)	*K_I_* (µM)
Verapamil [36] (CYP2C8)	0.065	17.45	0.082 ± 0.004	25 ± 4
Fluoxetine [36] (CYP2C8)	0.083	294	-	-
Isoniazid [36] (CYP2C8)	0.042	374	0.036 ± 0.003	280 ± 40
Phenelzine [36] (CYP2C8)	0.243	1.2	0.21 ± 0.02	0.84 ± 0.24
Nortriptyine [36] (CYP2C8)	0.036	49.9	0.044 ± 0.003	66 ± 12
Bergamottin [37] (CYP 3A4)	0.3	7.7	0.39 ± 0.05	12 ± 5
Tamoxifen [38] (CYP3A4)	0.04	0.2	0.043 ± 0.002	0.38 ± 0.12
N-desmethy-ltamoxifen [38] (CYP3A4)	0.08	2.6	0.083 ± 0.007	2.5 ± 1.0
Ethynyl-estradiol [39] (CYP3A4)	0.04	18	0.036 ± 0.008	5.4 ± 2.5

**Table 2:** IC_50_-values and kinetic parameters of reversible inhibitors interacting with HDAC8.

Cpd		IC_50_ (µM)	IC_50_ (µM) (Lit.)	*k_on_* × 10^3^ (M^−1^s^−1^)	*k_off_* × 10^−4^ (s^−1^)	*K_i_* (µM) *	*k_r_* × 10^−4^ (s^−1^)	*k_−r_* × 10^−4^ (s^−1^)
SAHA	Fast reversible	0.9 ± 0.2	1.9 [42]	-	-			
PCI-34051		0.11 ± 0.01	0.01 [43]	-	-	0.04		
SVE04		4.2 ± 1.1	-	-	-	1.3		
SVE27		2.6 ± 0.4	-	-	-	0.8		
mm182	Slow 1-step	0.16–1.3	0.31 [40]	2.3 ± 0.2	7.3 ± 0.8	0.31		
mm255		0.02–0.16	0.058 [40]	37 ± 2	13 ± 2	0.04		
mm220	Slow 2-step	0.19–1.88	0.14 [40]			2.6	40 ± 3	7.7 ± 0.7
SATFMK		0.02–0.10	0.021 [42]			0.14	180 ± 67	n.d.

* Binding constant for the formation of the protein–ligand encounter complex. The IC_50_ values of the slow-binding inhibitors change over time. n.d. means not determined.

**Table 3:** Kinetic parameters for covalent inactivators of HDAC8.

Cpd	IC_50_ (µM)	IC_50_ (µM) (Lit.)	*k_inact_*/*K_I_* (M^−1^s^−1^)	K_I_ (µM) *	*k_inact_* (min^−1^)
Ebselen	0.08 ± 0.03	-	n.d.	-	-
P2742	0.10 ± 0.02	0.11 [47]	n.d.	-	-
NEM	0.7–42	-	133 ± 3	-	-
TJ-19-24	0.4–4	-	2825	5.9 ± 0.8	1.0 ± 0.1
TJ-19-28	0.3–3	-	2500	12 ± 3	1.8 ± 0.2
SAH03	2.9–20	0.26 [46]	12,500	2.0 ± 1.3	1.5 ± 0.7

* Inactivator concentration with half-maximum *k_inact_*. The IC_50_ values for the slow-reacting inactivators change with time. n.d. means not determined.

## Data Availability

Data are contained within the article or Supplementary Materials.

## Supplementary Materials

The following supporting information can be downloaded at: https://www.mdpi.com/article/10.3390/ijms25115593/s1.
