# Supplementary material for: Rapid Determination of Kinetic Constants for Slow-Binding Inhibitors and Inactivators of Human Histone Deacetylase 8
Source: Int J Mol Sci. 2024 May 21;25(11):5593. doi: 10.3390/ijms25115593 (PMC11171933; doi:10.3390/ijms25115593)
Supplement: Supplementary file 1 [file ijms-25-05593-s001.zip › ijms-2999717-supplementary.pdf]

Supporting Information

Rapid Determination of Kinetic Constants for Slow-Binding Inhibitors and Inactivators of Human Histone Deacetylase 8

Aleksandra Kopranovic and Franz-Josef Meyer-Almes

Fig. S1: Reanalysis of literature data for mechanism-based inactivation of CYP enzymes

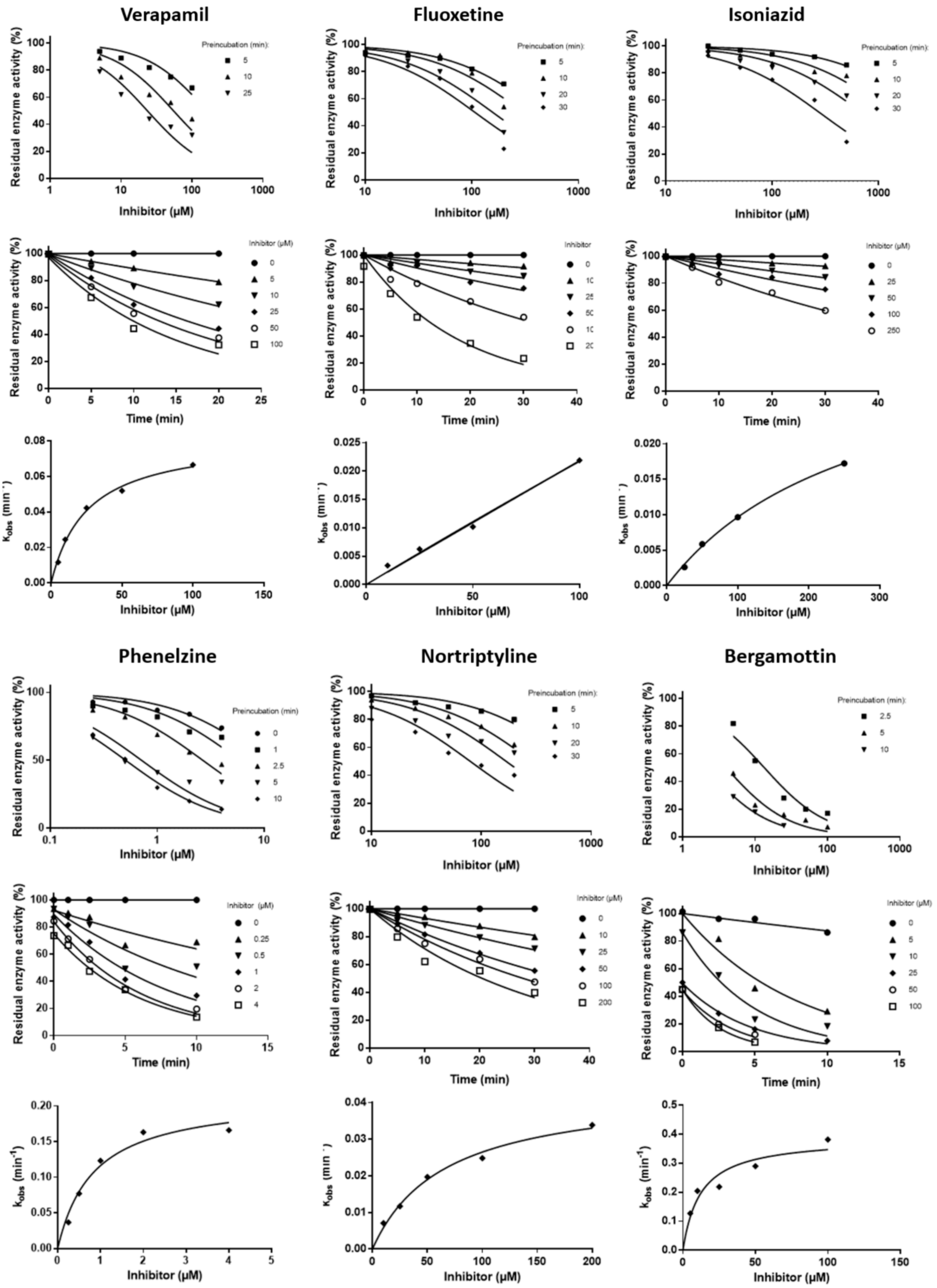

Figure S1: Reanalysis of literature data for mechanism-based inactivation of CYP enzymes by indicated active substances. Upper line shows dose response curves with different preincubation times, middle line shows the timedependent decay of relative enzyme activity in the prescencs of indicated inhibitor concentrations and the bottom line shows k_{obs} caculated from the decay curves above vs. inhibitor concentration.
